# Xerostomia (dry mouth) in patients with COVID-19: a case series

**DOI:** 10.2217/fvl-2020-0334

**Published:** 2021-04-06

**Authors:** Yaser Fathi, Elaheh Ghasemzadeh Hoseini, Fatemeh Atoof, Reza Mottaghi

**Affiliations:** 1 ^1^Department of Oral & Maxillofacial Medicine, DDs, MSc, Assistant Professor, Alborz University of Medical Sciences, Tehran, Alborz, Iran; 2 ^2^Department of Oral & Maxillofacial Medicine, DDs, MSc, Assistant professor, School of Dentistry, Kashan University of Medical Science, Isfahan, Iran; 3 ^3^Biostatistics, Assistant Professor, Kashan University of Medical Sciences, Kashan, Iran; 4 ^4^Department of Oral & Maxillofacial Surgery, DDs, student Dentistry, Student Research Center, School of Dentistry, Kashan University of Medical Sciences, Isfahan, Iran

**Keywords:** COVID-19, dry mouth, SARS-CoV-2, xerostomia

## Abstract

**Introduction:** Dry mouth has been reported as a symptom of COVID-19. In this study, xerostomia (dry mouth) was reported in patients with COVID-19. **Materials & methods:** Dry mouth was assessed in hospitalized patients with COVID-19 daily until all of the dry mouth symptoms resolved. **Results:** Dry mouth appeared in 60% of cases 3–4 days before as prodromal symptom and in others, simultaneously or 1–2 days after the onset of other symptoms. In most cases, with starting the treatment, dry mouth gradually disappeared. **Conclusion:** Xerostomia in COVID-19 could occur before the common symptoms. Therefore, it could be hypothesized that it could be used for early diagnosis, quarantine and treatment. As a result, disease transmission might be prevented and the best treatment outcomes could be achieved.

Dry mouth is a disorder that occurs due to insufficient saliva secretion or absolute salivary gland dysfunction. However, in many cases, this disorder is due to alterations in the quality of saliva, while the quantity of saliva does not vary much [1,2]. Drugs are the most common cause of dry mouth. Type 1 diabetes, hyperthyroidism, renal failure, vitamin deficiencies and some acute or chronic viral infections such as mumps, HIV and CMV are some of the other causes of dry mouth [1]. In recent months, following the outbreak of the new coronavirus pandemic, some cases of dry mouth related to COVID-19 have been reported which has attracted the attention of researchers.

COVID-19 is an acute respiratory syndrome caused by a beta-corona virus called coronavirus-2 (SARS-CoV-2) [3]. The virus, which has been spreading worldwide since the initial reports in China in December 2019, has infected more than 20 million individuals worldwide by 15 August 2020 and caused more than 700 thousand deaths [4]. Recent studies have shown that SARS-CoV-2 has structural differences from SARS-CoV and MERS. Hence, it displays a higher affinity for ACE2 receptors to enter host cells [3]. Extensive researches on these receptors have revealed that in addition to the common organs, ACE2 exists in various oral mucosal tissues, so, the oral cavity is considered a potential route for SARS-CoV-2 entry. ACE2 receptors are particularly observed in tongue and floor of the mouth, followed by the buccal mucosa and gingival epithelium [3]. Interestingly, studies have shown that these receptors are also present in salivary gland epithelial cells, and salivary gland cells are believed as one of the first SARS-CoV-2 target cells in primates [3]. Accordingly, it may be possible to gain major progress regarding the early diagnosis and treatment of individuals with COVID-19 by studying salivary changes and their associated oral symptoms. In the following, we present patients with COVID-19 who represented dry mouth as a primary complaint that sometimes continued over the course of the disease. In this study, xerostomia (dry mouth) was reported in patients with COVID-19.

## Materials & methods

Patients with COVID-19 were admitted to the Infectious Diseases Department of Shahid Beheshti Hospital in Kashan, Iran from mid-May to the end of June 2020. About 60% of the patients had dry mouth, which was more than the reported statistics in other studies (about 46%) [3].

In the present case series study, 10 patients with COVID-19 who had been examined in this center during this period and complained of dry mouth have been studied. The patients in this manuscript have given written informed consent to the publication of their case details and was reported after approval from the ethical committee in Kashan University on 11 August 2020 (ID: IR.KAUMS.REC.1399.025). Information on demographic characteristics, medical history, symptoms of the disease, diagnostic test results and treatment regimen of each patient were extracted from their hospital records. The state and severity of dry mouth were assessed via the visual analog scale (VAS) and were registered daily until all of the dry mouth symptoms resolved. To this end, patients were asked to rate the severity of dry mouth using a scoring system from 0 (for the absence of dry mouth) to 10 (for the most severe case of dry mouth). Thus, the changes in dry mouth symptoms were monitored and evaluated during the disease.

## Results

Demographic, diagnostic and therapeutic data of patients are summarized in Table 1. Out of ten cases, five were female and five were male. The mean age of the subjects was 42.63 years, with the oldest being 49 years old and the youngest 19 years old. Three of the subjects had gastrointestinal disorders and used proton pump inhibitors. Further, one of the subjects had an allergy and was taking antihistamines including hydroxyzine. One of the patients was a heavy smoker. However, all of these patients had never experienced dry mouth and had no complaints of symptoms before developing COVID-19 (it was the first time they had complained of dry mouth). Other patients had no medical history and did not take any specific medication. The most common symptoms at the time of admission were fever, cough, shortness of breath, fatigue and lethargy, although gastrointestinal symptoms and loss of taste were also reported in two cases. Diagnostic tests for COVID-19 included RT-PCR and CT scan of the lungs was carried out at the time of admission for all patients. The patients’ treatment regimens were comparatively similar and included antiviral drugs, hydroxychloroquine, antibiotics and oxygen supplementation. Based on their medical history, none of the patients maintained a history of radiotherapy or treatment with radioactive iodine of head and neck.

**Table 1.** Data of patients.

	P1	P2	P3	P4	P5	P6	P7	P8	P9	P10
Age	42	33	19	49	48	49	29	35	38	34
Gender	F	M	F	M	F	F	M	F	M	M
Medical history	_	_	Gastroesophageal reflux disease	_	Peptic ulcer disease (history)	PUD	_	_	_	Allergy
Medication	_	_	Pantoprazole	_	_	Omeprazole	_	_	_	Hydroxyzine
History of dry mouth	_	_	_	_	_	_	_	_	_	_
History of radiotherapy or iodine therapy	_	_	_	_	_	_	_	_	_	_
Smoking and/or alcohol	_	_	_	_	_	_	Smoking	_	_	_
Initial findings	Heartburn	Fever, cough	Fever, weakness, lethargy	Weakness, anorexia, cough	Dry cough, lethargy	Fever, shortness of breath	Shortness of breath	Weakness, lethargy	Shortness of breath, cough	Shortness of breath
Days from disease onset to xerostomia	Simultaneously with other symptoms	2 days before other symptoms	3 days before other symptoms	1 day after other symptoms	1 day before other symptoms	Simultaneously with other symptoms	1 day before other symptoms	3 days before other symptoms	Simultaneously with other symptoms	1 day before other symptoms
Imaging features	_	Multifocal	Multifocal	Multifocal	_	Multifocal	Bilateral involvement	Multifocal	_	_
Covid-19 treatment plan (drugs)	Atazanavir azithromycin vancomycin salbutamol heparin	Ribavirin heparin salbutamol prednisone	Ribavirin azithromycin ceftriaxone dextromethorphan	Meropenem heparin salbutamol promethazine IFN	Ceftriaxone heparin ribavirin amlodipine	Heparin salbutamol IFN ribavirin hydroxy- chloroquine meropenem	Salbutamol azithromycin IFN hydroxy- chloroquine meropenem	Heparin IFN hydroxy- chloroquine meropenem prednisone	Heparin salbutamol ribavirin hydroxy- chloroquine meropenem	Ceftriaxone loperamide apotel expectorant
Duration of hospitalization	10	7	3	3	7	10	6	3	7	5
Days from xerostomia onset to remission	7	7	7	2	2	18	5	17	16	8
VAS	6	5	8	8	6	5	7	5	5	7

Based on the information collected via the monitoring of dry mouth symptoms, four patients encountered slight dry mouth (VAS = 1–3), four patients experienced moderate dry mouth (VAS = 4–7), and only two patients experienced severe dry mouth (VAS = 8–10). Symptoms of dry mouth appeared in six patients (60% of cases) 3–4 days before the general infection symptoms such as fever and respiratory complications. In other patients, dry mouth was observed simultaneously or 1–2 days after the onset of other symptoms (including cough and shortness of breath). In patients who had started with general symptoms such as fever and respiratory symptoms, dry mouth symptoms such as dysphagia, foamy saliva, and dry lips were often observed at higher degrees of VAS. In all cases, with starting the treatment and recovery, the symptoms of dry mouth gradually decreased. Moreover, in most cases (eight patients), it completely disappeared after a few days (2–13 days). Only two patients still displayed slight degrees of dry mouth after almost three weeks (VAS = 1–2). In our hospital setting, negativization of PCR test was not considered as a criterion for the patient’s remission and discharge. Most of the patients were not tested again to investigate the negativization. Therefore, no data about oral indicators after negativization of PCR are provided. We recommend a comparison of VAS or xerostomia inventory between patients during pathology and the same patients after 1 month of negativization of RT-PCR. It should be useful to analyze the p-value.

## Discussion

A total of 10 COVID-19 patients with signs of xerostomia were evaluated in the present study. As mentioned earlier, none of the cases had experienced the symptoms of xerostomia before. In addition, none of the risk factors of xerostomia were noted in the medical history of these people. It was surprising that xerostomia in approximately 60% of the patients in our study began 1–4 days prior to hospitalization and receiving COVID-19 medications.

Freni *et al.* reported the signs of xerostomia in 32% of the COVID-19 patients in their investigation. These researchers demonstrated that in most of the cases, xerostomia occurred before other symptoms of the disease, and the severity of this problem diminished after 15 days [5,6]. The latter results are in line with our findings. Also, Fantozzi *et al.* reported xerostomia in 45.9% patients with median score of five (range: 3 to 8) and in 76.5% of patients their first-time experiencing xerostomia in their lifetime. According to this study xerostomia was reported as one of the first symptoms in 19.6% of patients with SARS-CoV-2 infection with a median onset time of 7 days (range: 4 to 7.8) before the COVID-19 diagnosis [6].

According to researches, SARS-CoV-2 uses ACE 2 receptors to enter the cells and utilizes the transmembrane serine protease Type 2 protein (TMPRSS 2) for priming [7]. The upregulation of these two receptors leads to the activation of the ribosomal pathway and the synthesis of viral RNA and proteins [8]. The ACE 2 and TMPRSS 2 receptors have a remarkable expression in the epithelial cells of salivary glands. Therefore, it seems that these glands are suitable receptors for SARS-CoV-2 and are among the first target cells of this virus and probably the virus can easily enter the salivary glands resulting in infection [8].

Different studies indicated that the SARS-CoV-2 virus is present in the whole saliva in the primary stages of the disease and is even detectable in the saliva secreted from salivary gland ducts after several days [3]. Liu *et al.* revealed that the SARS-CoV load was higher in saliva samples than blood specimens 48 h after intranasal viral challenge [9]. Considering the aforementioned data, virus penetration into salivary glands affects the function of these glands in the initial stages of the disease causing changes in saliva flow and components [3,10]. One of the theories proposed for the latter alterations is the neurotropic role of SARS-CoV-2 [5]. Coronavirus could enter the CNS through intranasal and peripheral nerves. Neurones and neuroglia; neural cells that express the entry protein ACE2 can be affected by coronavirus. Therefore, even occurrence of a neurological disease is possible. Furthermore, the involvement of SARS-CoV-2 in CNS infections was underscored by the findings that made use of transgenic mouse models expressing the human ACE2, which is the cellular receptor used by the SARS-CoV-2 to infect susceptible cells. The virus enters peripheral nerves and spreads to the CNS through synaptic contacts. Sensory and autonomic innervation of parotid gland and parasympathetic innervation of submandibular glands can be affected [5]. ACE2 receptors have been found in the epithelium of taste buds and salivary glands, not only in rhesus macaques but also in humans. Salivary glands in rhesus macaques have been demonstrated to be an early target for SARS-CoV-2 and SARS-CoV RNA has been demonstrated to be present in saliva before pulmonary lesions [11].

According to the literature, the neuropathic and mucotropic effects of this virus can potentially affect the function of salivary glands and lead to hyposalivation and xerostomia [12]. Moreover, inflammatory and infectious procedures have been noted as factors influencing reduced saliva. As a result, the possibility of quantitative and qualitative salivary disorders due to SARS-CoV-2 infection in the salivary gland should be taken into consideration [13].

Consuming numerous medicines and diverse pharmacological groups is among other reasons suggested for xerostomia in COVID-19. The most common medications in patients with COVID-19 include antiviral agents (remdesivir), hydroxychloroquine, anti-HIV medications (ritonavir, lopinavir) and interferons [12]. Overall, medications are the most frequent reason for xerostomia. Among the common medicines for COVID-19 treatment, lopinavir, ritonavir and interferons play a remarkable role in xerostomia. Studies showed that the xerogenic effects of some of these medications remain even after complete recovery from the disease [12]. However, we mentioned earlier that the signs of xerostomia in COVID-19 patients usually occur before other symptoms and treatment initiation and decrease gradually during the recovery period. The latter point reduces the possibility of the role of medicines in generating xerostomia. It was also notable in our study that the signs of xerostomia began simultaneously or a little after the incidence of COVID-19 symptoms in most cases (60%).

Lechien *et al.* investigated three cases of parotitis due to COVID-19 and mentioned lymphadenitis in the parotid gland as the reason. These authors demonstrated that elevation in parotid volume might result in the obstruction of Stensen duct, saliva retention and xerostomia due to hyposalivation [14]. However, none of the cases reported in the current study had signs of parotitis, such as a large parotid gland and the resultant obstruction of the salivary gland duct. Consequently, the exclusive report of dryness feeling by the patients is consistent with our results supporting the theory that the initial site of virus impact is salivary gland cells.

Knight *et al.* indicated that congestion and rhinorrhea in patients with COVID-19 could lead to mouth breathing accompanied by sequels, such as xerostomia [15]. Anxiety and worries related to being affected by disease and hospitalization might be considered as background factors for xerostomia in patients [15]. In the current investigation, as mentioned earlier, 60% of the cases had the feeling of dry mouth without other common symptoms and this early onset of xerostomia was interesting.

## Conclusion

One of the oral manifestations in COVID-19 patients is xerostomia or dry mouth. Although diverse reasons have been noted for xerostomia in these cases, it seems that the presence of SARS-CoV-2 virus in salivary glands and the resultant alterations in these glands could be the most important reason for this sign. Xerostomia usually occurs a little before the common symptoms of the disease, namely fever, cough and dyspnea. Therefore, it could be hypothesized that this sign could be used for early diagnosis, quarantine and treatment of the patients. As a result, disease transmission might be prevented and the best treatment outcomes could be achieved.

**Summary points**

- SARS-CoV-2 could travel to other tissues such as salivary glands and make some alterations to them resulting in oral manifestations.
- Xerostomia might be the initial manifestation of COVID-19 before signs and symptoms in the respiratory system.
- It is prudent to consider COVID-19 as a differential diagnosis in case of xerostomia.
- Saliva can be used for detection of SARS-CoV-2.
- Xerostomia symptom might be useful for early diagnosis, quarantine and treatment of the patients with SARS-CoV-2.
